# Time-domain adjoint optimization for metalens design toward enhanced broadband efficiency and uniformity

**DOI:** 10.1016/j.isci.2025.112739

**Published:** 2025-06-25

**Authors:** Mingyu Park, Haejun Chung, Kyung-Young Jung

**Affiliations:** 1 Department of Electronic Engineering, Hanyang University, Seoul 04763, South Korea; 2 Department of Artificial Intelligence, Hanyang University, Seoul 04763, South Korea

**Keywords:** Natural sciences, Optics, Applied sciences

## Abstract

We propose a time-domain adjoint optimization method for achromatic metalens design, achieving high efficiency and near-uniform spectral response. Unlike frequency-domain approaches, which require simulations for each sampled frequency, our method evaluates the entire frequency band continuously with consistent simulation times. Dynamically adjusting design variables and incident pulses during iterations balances performance and spectral uniformity. For a numerical aperture (NA) of 0.99, absolute focusing efficiency improves by 20%–30% over frequency-domain designs, with average efficiency increasing from 27% to 45%. Computational efficiency is demonstrated with 4.98 s per iteration compared to 61.8 s for frequency-domain methods. Scalability analysis shows high versatility across NAs (0.8, 0.6, and 0.4) and lens sizes (6.75 μm–50 μm), maintaining 70%–80% focusing efficiency with linear simulation time growth. This methodological advancement in nanophotonics establishes an efficient framework for designing high-performance optical devices with broad applications in imaging, communications, diagnostics, and precision metrology.

## Highlights

• Time-domain adjoint optimization enhances broadband metalens efficiency
• Dynamic pulse modulation improves spectral uniformity for designing metalens
• High-NA metalenses achieve improved achromatic performance with near-uniform response
• Gaussian-shaped pulses selectively target low-efficiency spectral regions

## Graphical abstract

**Figure:**
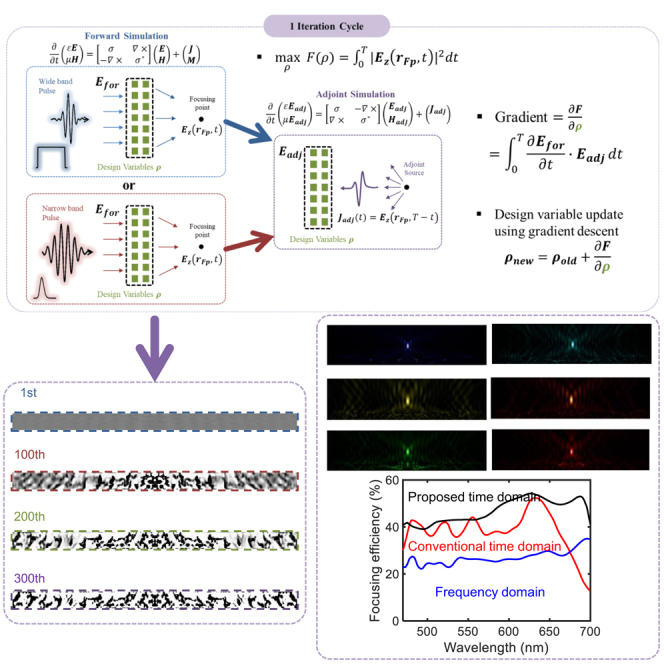

## Introduction

Metasurfaces^1^^,^^2^^,^^3^^,^^4^^,^^5^ composed of precisely engineered subwavelength nanostructures offer unprecedented control over the amplitude, phase, polarization, and other properties of light.^6^^,^^7^^,^^8^ This ability has fundamentally changed the interaction of light with matter, paving the way for innovative optical devices and applications. Metalenses,^9^^,^^10^^,^^11^^,^^12^ which are among the most promising metasurface applications, have enormous potential ranging from optical imaging^13^ and communications^14^ to biomedical diagnostics^15^ and optical metrology.^16^ Recent developments include wide-angle and high-efficiency metalenses designed using metagratings^17^ or angular-dispersionless metasurfaces,^18^^,^^19^ addressing key challenges such as narrow field of view and chromatic aberration. However, the optimal design of metalenses has relied on expert intuition and experience, often using methods such as unit cell approaches. However, these methods are limited by their local periodic assumptions, which can restrict exploration of the entire design space and lead to convergence on suboptimal solutions.^1^^,^^11^^,^^20^^,^^21^^,^^22^^,^^23^ To overcome the limitations of conventional design strategies, inverse design techniques have been developed to enable freeform nanophotonic structures with high performance.^24^^,^^25^^,^^26^^,^^27^^,^^28^ Recent developments also include applications in inverse-designed thin films,^29^ reconfigurable phase-change photonics,^30^ anisotropic material optimization,^31^ and slow-light nanostructures for ultrashort pulse manipulation.^32^ Among these, inverse design approaches based on adjoint methods—originally developed in mechanical engineering^33^ and control theory^34^—have emerged as powerful and efficient optimization tools.

Although adjoint methods are widely applied in various fields, such as mechanical engineering,^35^ fluid dynamics,^36^ and acoustics,^37^ they have shown particular promise in nanophotonics.^38^^,^^39^^,^^40^^,^^41^^,^^42^^,^^43^ The adjoint method enables efficient computation of gradients with respect to a large number of design parameters by performing two simulations: a forward simulation to solve the governing physical equations and an adjoint simulation to propagate objective-related sensitivities through the transposed system.^44^

Although some adjoint methods have proven effective, previous metalens designs have relied on frequency-domain formulations,^38^^,^^41^^,^^42^^,^^43^^,^^44^^,^^45^^,^^46^^,^^47^ which have significant limitations for broadband applications. These frequency-domain approaches necessitate individual simulations for each frequency component, making them computationally intensive and impractical for broadband applications. Recent advances have shifted the focus to time-domain adjoint methods using the finite-difference time-domain (FDTD) approach.^48^^,^^49^^,^^50^^,^^51^^,^^52^^,^^53^ FDTD method is particularly useful for broadband nanophotonic device optimization because it can efficiently handle wide frequency ranges and complex material properties.^54^^,^^55^^,^^56^^,^^57^ While pioneering works by Chung et al.^58^^,^^59^ and Nomura et al.^60^ established basic time-domain adjoint formulations, both used Gaussian pulses, which induced significant deviations within the operating frequency band. Yasuda et al.^61^ employed a time-domain adjoint method for metalens design, focusing on unit-cell-based optimization with group delay modifications. While their approach utilized level-set optimization to refine unit-cell morphologies, it could suffer from discrete sampling limitations and strong inter-cell coupling effects,^38^ leading to efficiency degradation in broadband applications. Lesina’s group^62^^,^^63^^,^^64^ developed an advanced framework specifically for metallic and dispersive structures, introducing truncated sinc functions to improve spectral distribution. Other researchers have explored pulse shaping^65^^,^^66^ and auto-differencing techniques,^67^ but time-domain adjoint optimizations continue to struggle with variations in performance over operating bandwidths, limiting the implementation of broadband nanophotonics.

In this article, we present a dynamic pulse-modulated time-domain adjoint method for designing achromatic metalenses with near uniform spectral response and high efficiency. Our approach involves three major contributions: a comprehensive analysis of existing frequency-domain^38^^,^^44^ and time-domain adjoint methods,^58^^,^^59^ introduction of an optimization technique that achieves both high efficiency and spectral uniformity, and detailed performance comparisons between frequency-domain and time-domain adjoint methods. We describe an achromatic metalens operating in the visible-light band that achieves the highest efficiency and lowest deviation reported in the literature, establishing a new benchmark for metalens design and nanophotonics.

Methodology of this paper introduces adjoint optimization techniques, beginning with frequency-domain method, followed by the conventional time-domain method, and culminating in the proposed approach. Results presents numerical examples of achromatic metalenses, offering a detailed comparison of the proposed method with conventional time-domain and frequency-domain adjoint methods,^38^ and discussing computational efficiency and scalability. Discussion and conclusion summarizes the key findings, highlights the implications of our approach, and proposes directions for future research.

## Methodology

The adjoint method computes the gradient of an objective function with respect to the design variables using two simulations: a forward simulation and an adjoint simulation. A major advantage of this method is its efficiency, as only two simulations are required to calculate the gradient of the objective function for all design variables. The forward simulation calculates the electromagnetic fields both within the design region and at the target area (where the objective function is defined). In the adjoint simulation, the forward fields related to the objective function are used as a source to compute adjoint fields. The gradient of the objective function with respect to each design variable is then obtained by combining these adjoint fields with the forward fields. For flexibility in the metasurface design, the design variable is defined as the permittivity of each cell within the design region (topology optimization^68^). The design variable $\rho \left(r_{\text{des}}\right)$ interpolates permittivity at location $r_{\text{des}}$ between two materials:

(Equation 1) $$\varepsilon_{r}\left(\rho \right)=\varepsilon_{1}+\left(\varepsilon_{2}-\varepsilon_{1}\right)\rho \left(r_{\text{des}}\right)\text{,}$$

where $0<\rho \left(r_{\text{des}}\right)<1$, and $\varepsilon_{1}$ and $\varepsilon_{2}$ represent the relative permittivity of the two materials. Here, $\rho \left(r_{\text{des}}\right)$ enables continuous permittivity adjustments between materials, and a penalty function enforces a binary design.^69^

### Frequency-domain adjoint method

The general objective function for the frequency-domain adjoint method is defined in^44^

(Equation 2) $$F_{f}=G\left(E,H\right)\text{,}$$

where $F_{f}$ is a real-valued objective function, defined as a function of the complex electric field E and the complex magnetic field H in the frequency domain. For time-harmonic electromagnetic fields, E and H, these complex field vectors are related to their instantaneous values through the time variation $e^{j\omega t}$. Specifically for metalenses, the general objective function (Equation 2) is modified to

(Equation 3) $$F_{f}=\frac{1}{2}{\left|E\left(r_{f}\right)\right|}^{2}=\frac{1}{2}E\;\cdot \;E^{*}\text{,}$$

where ∗ denotes the complex conjugate and $r_{f}$ represents the focal point. The gradient of $F_{f}$ with respect to the design variable *ρ* is given by

(Equation 4) $$\frac{\partial F_{f}}{\partial \rho }=\frac{1}{2}\left\{\frac{\partial E}{\partial \rho }\cdot E^{*}+\frac{\partial E^{*}}{\partial \rho }\cdot E\right\}=R\left\{\frac{\partial E}{\partial \rho }\cdot E^{*}\right\}\text{,}$$

where R represents the real part. To simplify the representation, we define $F^{\prime }$ as the inner term in Equation 4

(Equation 5) $$F^{\prime }=\frac{\partial E}{\partial \rho }\cdot E^{*}\text{.}$$

For time-harmonic Maxwell’s equations, the field relationships are expressed as

(Equation 6a) $$j\omega \mu H+\nabla \times E+M_{\text{src}}=0\text{,}$$

(Equation 6b) $$j\omega \varepsilon_{0}\varepsilon_{r}\left(\rho \right)E-\nabla \times H+J_{\text{src}}=0\text{,}$$

where *μ* is the permeability, and $M_{\text{src}}$ and $J_{\text{src}}$ represent the magnetic and electric current sources, respectively. Differentiating these equations with respect to *ρ* gives

(Equation 7a) $$j\omega \mu H_{\rho }+\nabla \times E_{\rho }=0\text{,}$$

(Equation 7b) $$\left(\varepsilon_{2}-\varepsilon_{1}\right)\varepsilon_{0}j\omega E+j\omega \varepsilon_{0}\varepsilon_{r}\left(\rho \right)E_{\rho }-\nabla \times H_{\rho }=0\text{.}$$

In the aforementioned equations, $E_{\rho }$ and $H_{\rho }$ represent the partial derivatives of E and H with respect to *ρ*, included for simplicity. By multiplying the adjoint fields $H_{\text{adj}}$ and $E_{\text{adj}}$ to (Equations 7a and 7b), respectively, and combining the two resulting equations with Equation 5, we obtain:

$$H_{\rho }\cdot \left[j\omega \mu H_{\text{adj}}+\nabla \times E_{\text{adj}}\right]$$

(Equation 8) $$+E_{\rho }\cdot \left[j\omega \varepsilon_{0}\varepsilon_{r}\left(\rho \right)E_{\text{adj}}-\nabla \times H_{\text{adj}}+E^{*}\right]$$

$$+F^{\prime }=\left(\varepsilon_{2}-\varepsilon_{1}\right)\varepsilon_{0}j\omega E\;\cdot \;E_{\text{adj}}\text{.}$$

To satisfy these equations, the terms in brackets must be zero, yielding following the adjoint system equations:

(Equation 9a) $$j\omega \mu H_{\text{adj}}+\nabla \times E_{\text{adj}}=0\text{,}$$

(Equation 9b) $$j\omega \varepsilon_{0}\varepsilon_{r}\left(\rho \right)E_{\text{adj}}-\nabla \times H_{\text{adj}}+J_{\text{adj}}=0\text{.}$$

Here, the adjoint current source $J_{\text{adj}}$ is defined as the complex conjugated electric field at the focal point in the forward simulation:

(Equation 10) $$J_{\text{adj}}\equiv E^{*}\text{.}$$

For a general objective function (Equation 2), the adjoint electric current source is given by

(Equation 11) $$J_{\text{adj}}=\frac{\partial G}{\partial E}\text{,}$$

and the adjoint magnetic current source is

(Equation 12) $$M_{\text{adj}}=\frac{\partial G}{\partial H}\text{.}$$

For the metalens design, the gradient of Equation 3 with respect to *ρ* can be summarized by substituting the last term of Equation 8 into Equation 4,

(Equation 13) $$\frac{\partial F_{f}}{\partial \rho }=R\left\{F^{\prime }\right\}=R\left\{\left(\varepsilon_{2}-\varepsilon_{1}\right)\varepsilon_{0}j\omega E\cdot E_{\text{adj}}\right\}\text{.}$$

For an achromatic metalens design using the frequency-domain adjoint technique, the objective function is extended to

(Equation 14) $$F_{f}\left(\rho \right)=\frac{1}{2}\sum_{i=1}^{N}{\left|E\left(\omega_{i},r_{p}\right)\right|}^{2}=\frac{1}{2}\sum_{i=1}^{N}E\left(\omega_{i},r_{p}\right)\;\cdot \;E^{*}\left(\omega_{i},r_{p}\right)\text{,}$$

where $E\left(\omega_{i},r_{p}\right)$ denotes the complex electric field at the focal point $r_{p}$ for each sampled frequency $\omega_{i}$. This formulation poses significant computational challenges due to the requirement for simulations that span a broad frequency range. During the forward simulations, the electric field $E\left(\omega_{i},r_{\text{des}}\right)$ is calculated for each frequency $\omega_{i}$. The adjoint sources for these simulations are defined as in Equation 10:

(Equation 15) $$J_{\text{adj}}=\sum_{i=1}^{N}\left|E\left(\omega_{i},r_{p}\right)\right|e^{-j\phi_{i}}\text{,}$$

where $\phi_{i}$ represents the phase of the electric field at frequency $\omega_{i}$. Using this adjoint source at each sampled frequency, the simplest and most straightforward method is to perform simulations separately for each frequency $\omega_{i}$. However, this is an inefficient approach due to the computational burden of simulating each frequency individually. To address this, two advanced methods can be used to incorporate the frequency-domain adjoint source into time-domain FDTD simulations.

In the multi-sinusoidal based method, the adjoint source is represented as a summation of sinusoidal functions as follows:

(Equation 16) $$J_{\text{adj}}\left(t\right)=\sum_{i=1}^{N}\left|E\left(\omega_{i},r_{p}\right)\right|\sin\left(\omega_{i}t-\phi_{i}\right)\text{.}$$

The adjoint sources are expressed as a sum of sinusoids, each with a magnitude and phase corresponding to the focal point, as determined in the forward simulation for each frequency. Once the sinusoidal responses for all frequencies reach a steady-state, additional simulation time is needed, with the total time proportional to the number of sampled frequencies. This is dictated by the number of samplings in the N-equations N-unknowns (NENU) method.^70^^,^^71^ The NENU approach requires longer computational time to convert the time-domain data into a frequency domain compared with a discrete Fourier transform (DFT), particularly when there are more than seven frequencies (or 35 when using Gaussian elimination via singular value decomposition).^70^

On the other hand, a pulse-based DFT method uses pulses to construct the adjoint source $J_{\text{adj}}\left(t\right)$:

(Equation 17) $$J_{\text{adj}}\left(t\right)=\sum_{i=1}^{N}\left|E\left(\omega_{i},r_{p}\right)\right|\sin\left(\omega_{i}t-\phi_{i}\right)\times e^{-{\left(t-t_{0}\right)}^{2}/t_{w}^{2}}\text{.}$$

In this expression, $t_{w}$ denotes the temporal width of the pulse, which must be sufficiently narrow to accurately resolve individual frequency components, and $t_{0}$ represents the delay time of the pulse. Using a pulsed source, a DFT is used to transform the time-domain data into the frequency domain. In broadband optimization, for which more than 20 frequency samples are typically used, DFT-based method is generally more computationally efficient than the NENU method. In both broadband frequency-domain adjoint simulations, the gradient of the objective function with respect to the design variable *ρ* is expressed as,

(Equation 18) $$\frac{\partial F_{f}}{\partial \rho }=\sum_{i=1}^{N}R\left\{\left(\varepsilon_{2}-\varepsilon_{1}\right)\varepsilon_{0}j\omega_{i}E\left(\omega_{i},r_{\text{des}}\right)\cdot E_{adj}\left(\omega_{i},r_{\text{des}}\right)\right\}\text{.}$$

All these methods are based on the frequency domain and therefore require extensive frequency sampling across a broad spectrum. This requirement makes them computationally burdensome for broadband device design. Moreover, their objective functions are structured at discontinuous sampled frequencies, posing significant challenges for practical wideband implementation.

### Conventional time-domain adjoint method

In contrast, time-domain adjoint methods offer a significant advantage over frequency-domain approaches, as the former operate in a continuous frequency spectrum (rather than at discrete sampled frequencies) and maintain a simulation time independent of the number of frequencies. This increases the efficiency of time-domain approaches for broadband device design. The objective function in time-domain adjoint methods is expressed as an integral, allowing it to account for the entire frequency spectrum continuously as follows:

(Equation 19) $$F_{t}=\frac{1}{2}\int_{0}^{T_{f}}E{\left(t,r_{p}\right)}^{2}\;dt\text{,}$$

where $T_{f}$ is the total time duration of the simulation. This integral formulation ensures that the entire frequency band is considered continuously, providing a more accurate and efficient method of evaluating broadband performance compared with frequency-domain methods that rely on discrete sampling. The gradient of the time-domain objective function with respect to the design variable is then given by

(Equation 20) $$\frac{\partial F_{t}}{\partial \rho }=\int_{0}^{T_{f}}E\left(t,r_{p}\right)\cdot \frac{\partial E\left(t,r_{p}\right)}{\partial \rho }\;dt\text{.}$$

Because (Equation 20) implies calculating the derivative for the electric field E with respect to all the design variables *ρ*, which is highly inefficient, we instead computed the derivative for each design variable directly in Maxwell’s equations using the adjoint method for simplification. For time-varying electromagnetic fields, Maxwell’s equations are expressed as

(Equation 21a) $$\mu \frac{\partial H}{\partial t}+\nabla \times E+M_{\text{src}}=0\text{,}$$

(Equation 21b) $$\varepsilon_{0}\varepsilon_{r}\left(\rho \right)\frac{\partial E}{\partial t}+\nabla \times H+J_{\text{src}}=0\text{.}$$

The differentiation of Equations 21a and 21b with respect to the design variables yields

(Equation 22a) $$\mu \frac{\partial H_{\rho }}{\partial t}+\nabla \times E_{\rho }=0\text{,}$$

(22b) $$\left(\varepsilon_{2}-\varepsilon_{1}\right)\varepsilon_{0}\frac{\partial E}{\partial t}+\varepsilon_{0}\varepsilon_{r}\left(\rho \right)\frac{\partial E_{\rho }}{\partial t}-\nabla \times H_{\rho }=0\text{.}$$

By multiplying (Equations 22a and 22b) by the adjoint vectors $H_{\text{adj}}$ and $E_{\text{adj}}$, respectively, and integrating over time for the entire duration, we obtain

(Equation 23a) $$\int_{0}^{T_{f}}\;\mu \frac{\partial H_{\rho }}{\partial t}\cdot H_{\text{adj}}+\nabla \times E_{\rho }\cdot H_{\text{adj}}\;dt=0\text{,}$$

(Equation 23b) $$\int_{0}^{T_{f}}\left(\varepsilon_{2}-\varepsilon_{1}\right)\varepsilon_{0}\frac{\partial E}{\partial t}\cdot E_{\text{adj}}+\varepsilon_{0}\varepsilon_{r}\left(\rho \right)\frac{\partial E_{\rho }}{\partial t}\cdot E_{\text{adj}}-\nabla \times H_{\rho }\cdot E_{\text{adj}}\;dt=0\text{.}$$

The time-derivative terms $\mu \frac{\partial H_{\rho }}{\partial t}H_{\text{adj}}$ and $\varepsilon_{0}\varepsilon_{r}\left(\rho \right)\frac{\partial E_{\rho }}{\partial t}E_{\text{adj}}$ can be simplified by using partial integration and applying a terminal condition on the adjoint vector $E_{\text{adj}}\left(t=T_{f}\right)=0,H_{\text{adj}}\left(t=T_{f}\right)=0$.

(Equation 24a) $$\int_{0}^{T_{f}}\;\mu \frac{\partial H_{\text{adj}}}{\partial t}\cdot H_{\rho }+\nabla \times E_{\rho }\cdot H_{\text{adj}}\;dt=0\text{,}$$

(Equation 24b) $$\int_{0}^{T_{f}}\left(\varepsilon_{2}-\varepsilon_{1}\right)\varepsilon_{0}\frac{\partial E}{\partial t}\cdot E_{\text{adj}}+\varepsilon_{0}\varepsilon_{r}\left(\rho \right)\frac{\partial E_{\text{adj}}}{\partial t}\cdot E_{\rho }-\nabla \times H_{\rho }\cdot E_{\text{adj}}\;dt=0\text{.}$$

By adding (Equations 20, 24a, and 24b), we obtain

$$\int_{0}^{T_{f}}\left[\mu \frac{\partial H_{\text{adj}}}{\partial t}-\nabla \times E_{\text{adj}}\right]\cdot H_{\rho }\;dt$$

(Equation 25) $$+\int_{0}^{T_{f}}\left[\varepsilon_{0}\varepsilon_{r}\left(\rho \right)\frac{\partial E_{\text{adj}}}{\partial t}+\nabla \times H_{\text{adj}}-E\left(t,r_{p}\right)\right]\cdot E_{\rho }\;dt$$

$$+\frac{\partial F_{t}}{\partial \rho }=-\int_{0}^{T_{f}}\left(\varepsilon_{2}-\varepsilon_{1}\right)\varepsilon_{0}\frac{\partial E}{\partial t}\;\cdot \;E_{\text{adj}}\;dt\text{.}$$

We note that Equation 25 is satisfied, if the following equations hold

(Equation 26a) $$\mu \frac{\partial H_{\text{adj}}}{\partial t}-\nabla \times E_{\text{adj}}=0\text{,}$$

(Equation 26b) $$\varepsilon_{0}\varepsilon_{r}\left(\rho \right)\frac{\partial E_{\text{adj}}}{\partial t}+\nabla \times H_{\text{adj}}-E\left(t,r_{p}\right)=0\text{,}$$

and the gradient of the objective function satisfies

(Equation 27) $$\frac{\partial F_{t}}{\partial \rho }=-\int_{0}^{T_{f}}\left(\varepsilon_{2}-\varepsilon_{1}\right)\varepsilon_{0}\frac{\partial E}{\partial t}\;\cdot \;E_{\text{adj}}\;dt\text{.}$$

Therefore, (Equations 26a and 26b) can be considered as adjoint system equations and are expressed in reverse time, subject to termination conditions, to resemble the classical Maxwell’s equations:

(Equation 28a) $$\mu \frac{\partial H_{\text{adj}}}{\partial \tau }+\nabla \times E_{\text{adj}}=0\text{,}$$

(Equation 28b) $$\varepsilon_{0}\varepsilon_{r}\left(\rho \right)\frac{\partial E_{\text{adj}}}{\partial \tau }-\nabla \times H_{\text{adj}}-J_{\text{adj}}=0\text{.}$$

Here, the adjoint source radiates the electromagnetic field, computed from the forward simulation at the focal point, in reverse time to calculate the gradient of the objective function with respect to the design variables:

(Equation 29) $$J_{\text{adj}}\left(t\right)\equiv E\left(\tau ,r_{p}\right)\text{.}$$

Note that adjoint source varies with the objective function. For a generalized objective function $F_{t}=\int_{0}^{T_{f}}G\left(E,H\right)\;dt$, the adjoint source is given by^59^

$$J_{\text{adj}}=\frac{\partial G\left(E,H\right)}{\partial E}\text{,}$$

$$M_{\text{adj}}=\frac{\partial G\left(E,H\right)}{\partial H}\text{,}$$

where, $M_{\text{adj}}$ is the adjoint magnetic current source. This formulation can be derived by applying the generalized objective function in place of Equation 19.

Time-domain adjoint methods offer significant advantages in broadband optimization over their frequency-domain counterparts. Unlike frequency-domain approaches, time-domain methods maintain consistent simulation times regardless of the number of frequencies under consideration. This efficiency arises from their ability to evaluate an entire frequency band in a single simulation, eliminating the need to process each frequency individually. The continuous consideration of the entire frequency spectrum in time-domain methods provides a wealth of information for more accurate calculations. This comprehensive approach often results in designs with superior overall performance, as measured by metrics such as average efficiency and sum over frequencies.

However, conventional time-domain approaches have drawbacks, as studies revealed a trade-off in optimized designs with time-domain methods.^58^^,^^59^^,^^60^^,^^61^^,^^62^^,^^63^^,^^64^ While these designs often exhibit high overall performance, they can suffer from significant deviations in the frequency band of interest. This phenomenon arises because the optimization process may sacrifice performance in certain frequency ranges to enhance the total integration (19), resulting in non-uniform performance across the entire bandwidth. This trade-off between overall performance and spectral uniformity presents an ongoing challenge in the field of broadband nanophotonics optimization, emphasizing the need for advanced techniques that balance these competing factors.

### Proposed time-domain adjoint method

**Figure 1:**
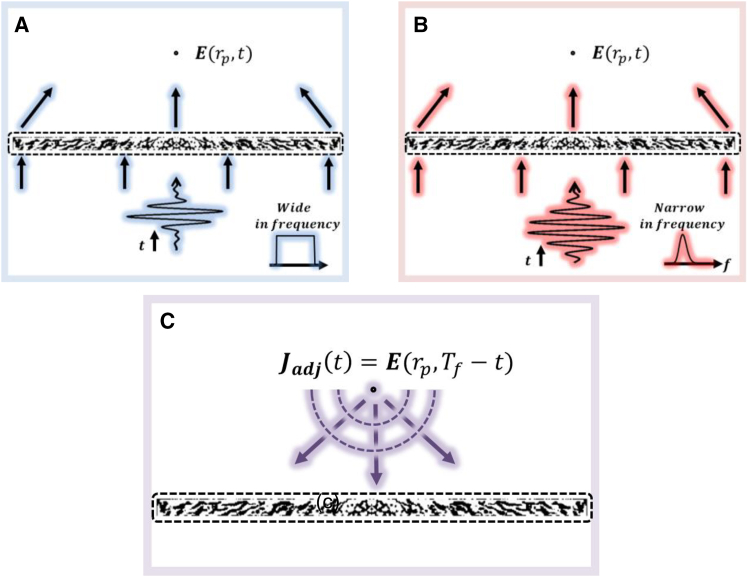
Schematic of the proposed time-domain adjoint method (A) Forward simulation with wideband sinc function incidence. (B) Modified forward simulation with narrow-band Gaussian incidence. (C) Adjoint simulation.

To address the shortcomings of conventional time-domain methods, we propose a methodology that dynamically adjusts not only the design variables but also incident pulse components in forward simulation of each iteration. Figure 1 illustrates the key aspects of our approach, highlighting operation of the proposed method through different iterations to achieve optimal broadband performance. Figure 1A depicts the initial forward simulation of our optimization process, which is also used in conventional time-domain methods. The incident pulse is a sinc function, represented by a broad waveform in the time domain and a rectangular spectrum in the frequency domain.^64^ The incident pulse with relatively uniform power distribution over the bandwidth allows an accurate optimization across all operating frequencies without applying weighting functions while also allowing for performance evaluation over a wide range of frequencies in the first iteration.

Even when aiming for the design of the achromatic metalenses, certain frequencies may exhibit degraded performance as the process progresses.

To address this, our proposed method identifies underperforming frequency and subsequently adjusts the incident pulse in later iterations, as illustrated in Figure 1B.

This case employs a narrow-band incident pulse, enabling focused optimization in specific frequency ranges where performance improvements are needed. Figure 1B does not depict a separate method but a subsequent iteration of our proposed approach, demonstrating its ability to dynamically adjust pulses to address performance issues identified in previous iterations. Throughout this process, whether using wideband pulses as shown in Figure 1A or narrow-band pulses as in Figure 1 (b), our method uses the same adjoint equation, illustrated in Figure 1C and as formulated in Equations 28a and 28b, with the adjoint source defined as in Equation 29.

In practice, our optimization typically starts with a broadband sinc function as the initial incident wave, as depicted in Figure 1A. The sinc function can be expressed as:

$$s\left(t\right)=\left\{ \begin{array}{cc} \frac{\cos\left(2\pi f_{0}t\right)\sin\left(2\pi f_{\text{BW}}\left(t-t_{0}\right)\right)}{2\pi f_{\text{BW}}\left(t-t_{0}\right)}B\left(t\right), & t\neq t_{0}\\ \cos\left(2\pi f_{0}t\right)B\left(t\right), & t=t_{0} \end{array}\right.$$

where the Hamming window B(t) is defined as:

$$B\left(t\right)=0.54-0.46\;\cos\left(\frac{2\pi t}{2t_{0}}\right),0<t<2t_{0}$$

Here, $f_{0}$ and $f_{\text{BW}}$ represent the center frequency and bandwidth at which the device operates, respectively, and B(t) serves as the Hamming window to modulate the signal.

As the algorithm identifies particular frequency with degraded performance, it shifts toward using more focused Gaussian pulses centered on that frequency bands, as depicted in Figure 1B. The Gaussian pulse is defined as:

$$s\left(t\right)=\exp\;\left(-\frac{{\left(t-t_{0}\right)}^{2}}{2\sigma^{2}}\right)\cos\left(2\pi f_{\min}t\right)\text{,}$$

where $f_{\min}$ denotes the frequency at which the focusing efficiency is lowest and *σ* controls the width corresponding to approximately 10% of $f_{\min}$. The frequency imbalance introduced in former iterations using a sinc function as the initial incident wave is effectively compensated by subsequently adopting Gaussian pulses. These pulses, with amplitudes concentrated on the central frequency and smoothly decaying bell-shape, allow the optimization process to selectively focus on frequency bands exhibiting the lowest efficiency. By intensively refining performance in these underperforming regions, this strategy significantly improves both spectral uniformity and overall efficiency. This dynamic adjustment of the pulse over the iterations enables the proposed method to progressively improve the frequency response across the entire operating band. By alternating between broadband and narrowband incident pulses, our approach exploits the broad coverage of broadband excitation and the spectral precision of narrowband tuning. As a result, the method facilitates the design of metalenses that maintain high and nearly uniform performance over the entire spectral range of interest.

The flexibility of our approach, transitioning as needed from the scenario in Figure 1A to that in Figure 1B while consistently applying the adjoint method shown in Figure 1C, ensures a more effective optimization process. This methodology paves the way for the design of high-performance, achromatic metalenses with consistent efficiency across their operational spectra, addressing the limitations of conventional time-domain optimization approaches.

## Results

### Verification of the time-domain adjoint method

In this study, we designed a broadband achromatic metalens operating in the visible-light spectrum (470–700 nm), using the proposed time-domain adjoint method and the conventional adjoint time-domain method, and compared the result with those of a metalens designed using the frequency-domain adjoint method.^38^ All simulations were performed on a workstation equipped with an Intel Core i7-12700 processor. First, the metalens design focused on a numerical aperture (NA) of 0.99, which is challenging due to the complexity of controlling the light phase caused by the short focal length. Titanium dioxide was chosen for the metalens as it exhibits lossless property in the visible-light region and has a dielectric constant of approximately 6. The metalens dimensions were set to have 12.5 μm in width and 500 nm in thickness, with a focal length of 900 nm to achieve the desired NA of 0.99. Each design cell measured 25 nm, enabling precise control of the design parameters. Also the FDTD grid size was set to 25 nm, ensuring consistency with the design resolution. For simulation stability, the Courant-Friedrichs-Lewy number^72^^,^^73^ was set to 0.99. A total-field/scattered-field technique^74^^,^^75^ was chosen to introduce a plane wave into the design region, while the edges of the design were wrapped with perfectly matched layers^76^^,^^77^^,^^78^ to simulate an unbounded region.

**Figure 2:**
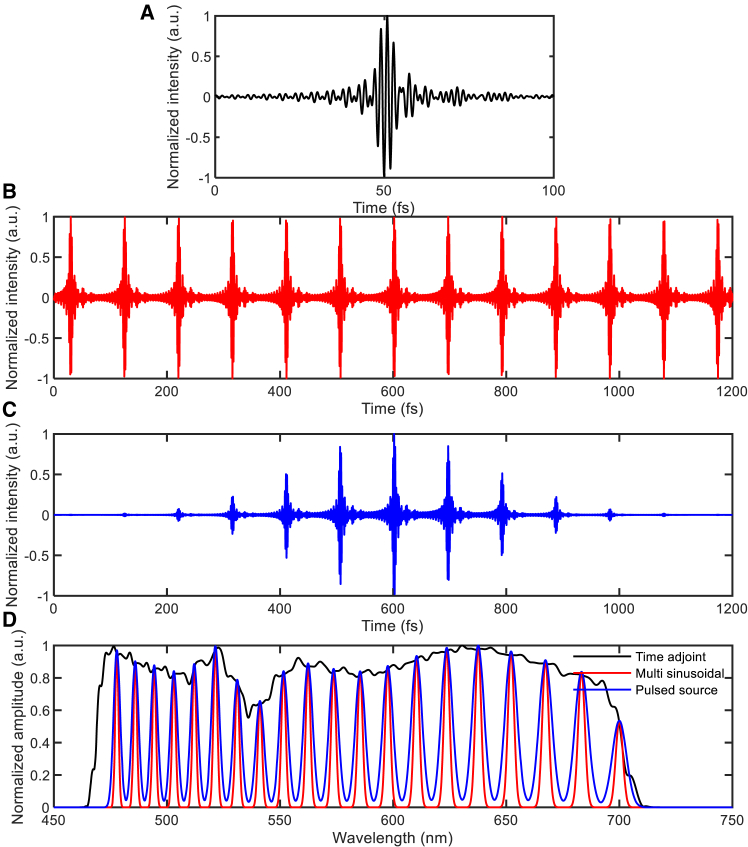
Comparison of adjoint simulation sources and their frequency components (A) Adjoint source used in the time-domain adjoint methods. (B) Multiple sinusoidal signals of the frequency-domain adjoint method. (C) Pulsed signals of the frequency-domain adjoint method. (D) Frequency components of the adjoint sources used in the three methods, showing equal amplitude at sampled frequencies.

To evaluate the design time of each introduced method, we first identified the adjoint simulation sources that contributed the most to the overall design time. For this evaluation, we considered a method that used the sinc function in the time-domain adjoint method, as well as two sources—the NENU-based multi-sinusoidal source (16) and the DFT-based pulsed source (17)—employed in the frequency-domain adjoint method. Each method used a broadband forward simulation; however, during the adjoint simulation, the time-domain method used the electric field at the focal point in reverse time (29), whereas the frequency-domain methods used multi-sinusoidal or pulsed sources that matched the magnitude and phase of the forward focal field. Figure 2A illustrates the adjoint source used in the time-domain methods. This source is the time-reversed electric field at the focal point of the forward simulation, in which a sinc function was used as the incident pulse. The total pulse duration of this adjoint source is confined to 100 fs. Figure 2B depicts the adjoint source constructed using multi-sinusoidal signals in the frequency-domain method, sampled at 20 frequency points within the same band. The amplitudes at the sampled frequencies are matched to those of the time-domain adjoint source. This method requires more than 1,200 fs for the response to reach a steady state, along with additional simulation time under NENU conditions.^70^ Figure 2C displays the pulsed adjoint signals corresponding to the same frequency sampling used in Figure 2B. This approach also requires approximately 1,200 fs—about 12 times longer than the time-domain method. Figure 2D presents the frequency components of the adjoint sources used in the three methods, each constructed to match the amplitude at the sampled frequencies with the time-reversed signal from the forward simulation. Although multiple time-domain envelopes can correspond to the same frequency-domain spectrum, this does not affect the solution space because the solution space mainly depends on the number of design variables and the imposed design constraints, which are conserved in time-domain or frequency-domain adjoint optimizations.

Next, we addressed the computation time per iteration. For the time-domain methods, the computation time per iteration was 4.98 s. For frequency-domain methods, the iteration time depends on the number of sampled frequencies and increases accordingly. With 20 frequencies, the multi-sinusoidal approach required 61.8 s, while the pulsed approach took 28 s. For 25 frequencies, the times increased to 76 s and 33 s, respectively. At 30 frequencies, the computation times were 91 s for the multi-sinusoidal approach and 39 s for the pulsed approach. This variation in simulation time between the multi-sinusoidal and pulsed methods stems from the computational burden of frequency-domain conversion, with the NENU method requiring more intensive calculations compared with the DFT-based approach. These results demonstrate the significant time advantage of the time-domain methods over its frequency-domain counterparts, particularly as the number of sampled frequencies increase. The proposed and conventional time-domain methods have identical design times due to their identical systems.

**Figure 3:**
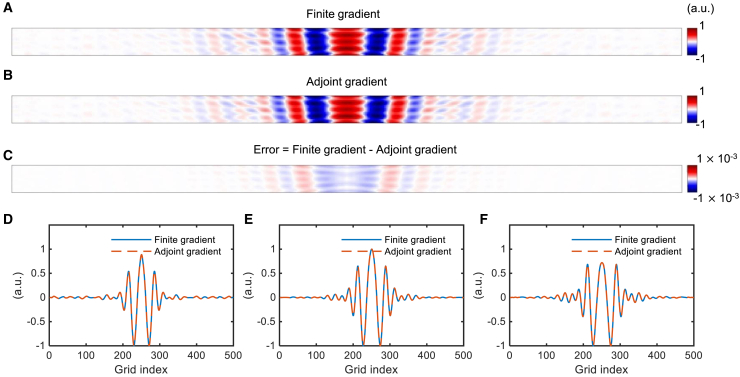
Verification of the time-domain adjoint method through gradient comparison (A) Normalized finite gradient. (B) Normalized adjoint gradient. (C) Error between finite and adjoint gradients. (D–F) Comparison of adjoint gradient and finite gradient values along the top, center, and bottom lines of the design region, respectively.

To verify the accuracy and tractability of the proposed time-domain method, we compared the computed adjoint gradient and finite gradient. Figure 3A shows the normalized value of the objective function’s gradient (finite gradient), which was calculated by slightly altering the design variables of each cell in each design region. Figure 3B dipicts the normalized adjoint gradient for the entire design region’s variables obtained through two simulations. The difference between them, with errors shown in Figure 3C, has a maximum value of $8.75\times 10^{-4}$, which is less than 0.1%. Figures 3D–3F compares the values of the top, center, and bottom lines of the adjoint gradient and the finite gradient, respectively, all of which were consistent.

**Figure 4:**
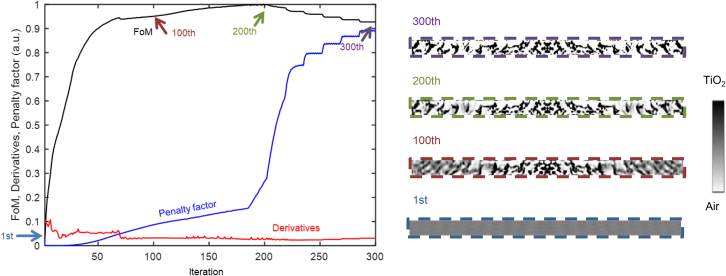
Optimization process The figure of merit, penalty factor, and derivatives over 300 iterations.

After verifying the accuracy of the proposed adjoint method, we examined the design iteration using a gradient-based optimization algorithm. In Figure 4, a sharp increase in the objective function, also called the figure of merit (FoM), can be seen, with high-derivative values up to approximately 50 iterations, after which the increase slows and the penalty factor ($1-\Sigma 4\rho \left(1-\rho \right)/N_{\rho }$)^43^ increases as the design becomes binarized. At the 75^th^ iteration, the penalty factor and derivatives intersect, causing a slight decrease in the FoM, which then slowly increases to its maximum at the 200^th^ iteration. The penalty factor subsequently increases sharply and the FoM decreases slightly, ultimately resulting in a perfect binarization into two materials, TiO_2_ and air.

**Figure 5:**
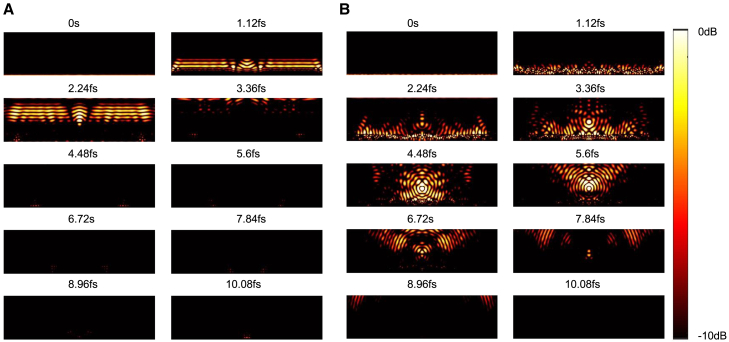
Snapshots of the metalens simulation (A) First iteration showing minimal focusing, behaving similarly to a plane wave. (B) 300^th^ iteration demonstrating effective phase change from the lens edge to the focal point, with most power concentrated near the focus. The scale bar means the intensity of the electric fields in dB scale.

Examining the simulation snapshots of the metalens in Figure 5A, it is clear that, in the first iteration, only a small fraction of the power passed through the focusing point $r_{p}$, behaving almost identically to a plane wave. However, as in Figure 5B, after the 300^th^ iteration, the phase changed from the outermost angle of the metalens to the focal point, with almost all power passing through and near the focal point, performing as a lens with high efficiencies.

### Broadband achromatic metalens operating in the visible light

**Figure 6:**
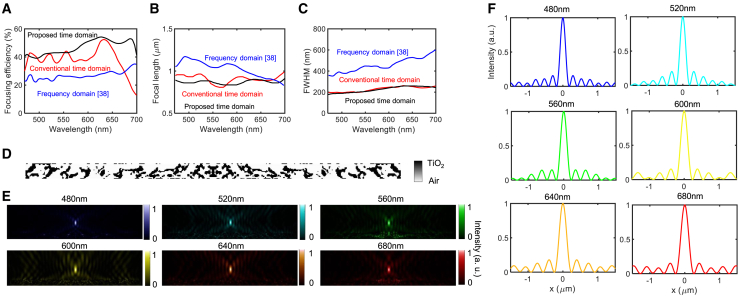
Analysis of metalens with an NA of 0.99 (A) Focusing efficiency for the proposed metalens, conventional time-domain design, and frequency-domain design.^38^ (B) Focal length. (C) FWHM. (D) Metalens design. (E) Field intensity distributions. (F) Intensity profiles.

The performance of the designed metalens at the 300^th^ iteration (Figure 6) was superior to those of both conventional time-domain and frequency-domain designs.^38^ We adopted the focusing efficiency definition from^79^—the ratio of the total energy enclosed within the focal spot, defined by the first minimum in the intensity profile, to the total incident energy. Our analysis reveals that a metalens designed by the proposed method achieves a 20%–30% increase in absolute focusing efficiency across all frequencies compared with a frequency-domain design^38^ and a 10%–15% improvement over the conventional time-domain method. The proposed method also achieves more consistent spectral uniformity compared with the significant deviations observed in conventional time-domain approaches, as seen in Figure 6A. The average efficiency improved from 27% for frequency-domain methods and 38% for conventional time-domain methods to 47% with our proposed method. The focal length profile in Figure 6B shows that the proposed design maintains values close to the design target of 0.9 μm, demonstrating that our method effectively suppresses chromatic dispersion across the operating wavelength range. The full width at half maximum (FWHM) of the metalens, shown in Figure 6C, reveals that the proposed method yields a narrower focal spot compared with frequency-domain methods and almost the same as that of the conventional method. Notably, for high-NA metalenses such as NA = 0.80, 0.99, the ideal intensity profile at the focal point deviates against the diffraction-limited focusing profile. This is because the paraxial approximation breaks down due to large deflected angles and strong vectorial effects at the lens edges.^80^^,^^81^ Recent studies have shown that high-NA metalenses can exhibit sub-Airy spot sizes and strong longitudinal field components, which are not captured by simple scalar diffraction models.^80^^,^^81^ Therefore, in this work, we do not compare the intensity profile against the theoretical one at high-NA regimes.

The binary topology design, depicted in Figure 6D, indicates that a high degree of freedom enables high performance. The intensity distributions for representative wavelengths in Figure 6E and the focal plane profiles in Figure 6F further confirm the superior focusing capabilities and minimal sidelobes of the designs produced by the proposed method. Overall, the results presented in Figure 6 show relatively low focusing efficiencies in frequency-domain methods, as well as the significant spectral non-uniformity observed in conventional time-domain approaches. In contrast, the proposed method demonstrates both improved efficiency and spectral uniformity, establishing it as a more effective strategy for achromatic metalens optimization.

In addition to metalenses with an NA of 0.99 designed for close-range focusing, our time-domain inverse design method demonstrates versatility in designing lenses with various focal lengths and numerical apertures. We designed and analyzed metalenses with NA values of 0.8, 0.6, and 0.4.

**Figure 7:**
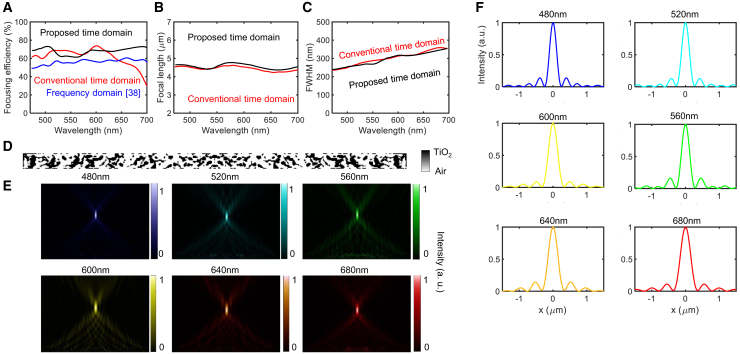
Analysis of a metalens with NA 0.8 (A) Focusing efficiency for the proposed metalens, conventional time-domain design, and frequency-domain design.^38^ (B) Focal length. (C) FWHM. (D) Metalens design. (E) Field intensity distributions. (F) Intensity profiles.

For the NA 0.8 metalens design, Figure 7A shows absolute efficiency improvements of 10%–20% compared with frequency-domain design results and significant improvement at 700 nm wavelength compared to the conventional time-domain method. The focal length profile in Figure 7B shows slight fluctuations around the design target of 4.75*μ*m for both the proposed and conventional methods, with the proposed design remaining closer to the target. Figure 7C shows that the FWHM increases linearly from approximately 250 nm to 350 nm. Figure 7D displays the binary topological design result, with Figures 7E and 7F showing field distributions across the visible spectrum in both the entire plane and the focal plane, respectively, demonstrating effective power concentration. In Figure 7, the conventional time-domain method outperforms the frequency-domain approach in overall focusing efficiency. However, the conventional time-domain method does not show a strong spectral uniformity, particularly in the focusing efficiency. In contrast, the proposed method not only achieves higher average efficiency but also maintains a more uniform spectral response, effectively addressing the non-uniformity seen in conventional designs.

**Figure 8:**
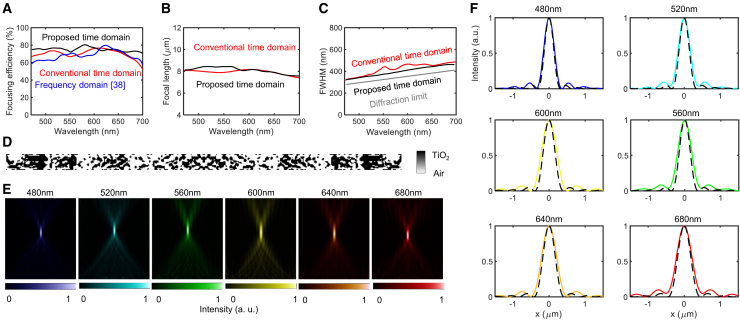
Analysis of a metalens with NA 0.6 (A) Focusing efficiency for the proposed metalens, conventional time-domain design, and frequency-domain design.^38^ (B) Focal length. (C) FWHM comparison with a diffraction-limited one. (D) Metalens design. (E) Field intensity distributions. (F) Normalized focal plane intensity profiles with diffraction-limited one (dashed line).

The NA 0.6 design had a sufficient focal length to assume a far-field approximation, comparable to that of a diffraction-limited system.^82^ In Figures 8A a metalens with an NA of 0.6 achieves higher efficiency than those of the NA 0.99 and NA 0.8 designs as the focal length increases, indicating that the optimization problem becomes easier to solve. Compared with the conventional time-domain and frequency-domain approaches, the performance maintains overall higher efficiency with low variance across the frequency band, while the conventional time-domain method shows slightly higher efficiency compared with the frequency-domain method. The focal length in Figure 8B remains stable at the design target of 8.325 μm for both the proposed and conventional time-domain methods. Figure 8C depicts FWHM values close to the diffraction limit, demonstrating that the proposed time-domain adjoint method approaches the theoretical performance boundary, whereas the conventional time-domain method exhibits overall larger FWHM values. Figure 8D illustrates the binarized topology design of the metalens, while Figures 8E and 8F depicts the focused power distribution, reaching the diffraction limit (indicated by dashed lines).

In this case, however, the performance gap between the proposed and conventional methods is relatively small. Unlike in the NA 0.99 case, where chromatic aberration is more severe due to a shorter focal length and larger diffraction angle, the relatively lower NA metalenses (e.g., NA 0.6, NA 0.4) show a naturally smaller angular spread and weaker chromatic sensitivity. As a result, the conventional time-domain method achieves near-optimal performance in mid to lower NA metalens designs, thereby, our proposed method can have marginal improvement compared to the other methods.

**Figure 9:**
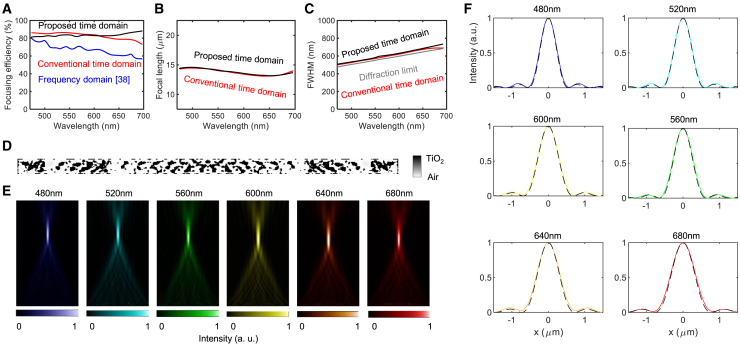
Analysis of a metalens with NA 0.4 (A) Focusing efficiency for the proposed metalens, conventional time-domain design, and frequency-domain design.^38^ (B) Focal length. (C) FWHM comparison with diffraction-limited one. (D) Metalens design. (E) Field intensity distributions. (F) Normalized focal plane intensity profiles with diffraction-limited one (dashed line).

The NA 0.4 design in Figure 9 follows similar trends, showing further improved efficiency with decreased NA while maintaining superiority over frequency-domain designs. The optimization problem becomes sufficiently straightforward as the focal length increases, such that the results of the conventional time-domain method are almost identical to those of the proposed method. The focal length in Figure 9B remains constant at 14.325 μm for both the proposed and conventional methods, with FWHM values also approaching the diffraction limit. The binary design pattern is depicted in Figure 9D. Field distributions in Figures 9E and 9F are consistent with focusing performances across the visible spectrum, reaching the diffraction limit (indicated by the dashed lines).

At this low NA regime, the optical system inherently exhibits minimal chromatic dispersion due to the long focal length and small diffraction angle. As such, even without proposed method, the conventional time-domain method already delivers near-optimal broadband performance. Therefore, the advantage of our proposed method becomes marginal in this setting, reinforcing that the most significant benefits of our approach emerge in high-NA, chromatically sensitive scenarios.

**Figure 10:**
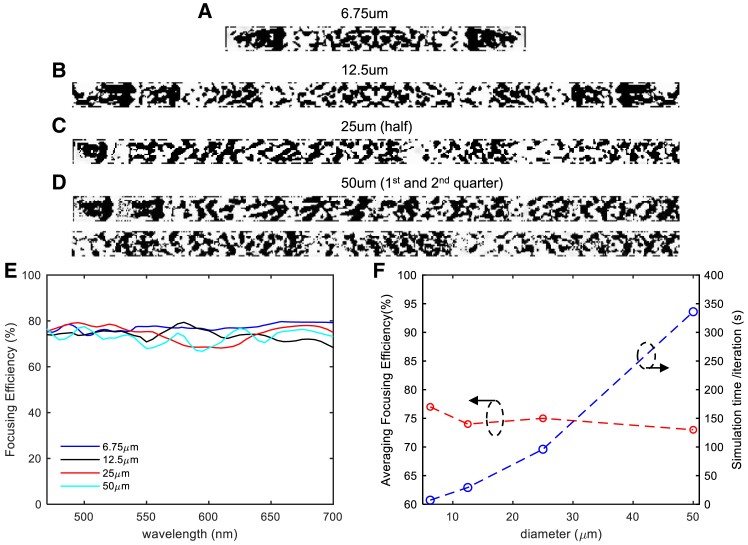
Scalability analysis of metalens designs with an NA of 0.6 (A–D) Designed metalens: (A) 6.75 μm width, (B) 12.5 μm width (Figure 8), (C) half of the 25 μm width design (symmetric), and (D) half of the 50 μm width design (symmetric). (E) Focusing efficiency. (F) Average focusing efficiency and simulation time per iteration.

To investigate design variable scalability, we conducted additional studies using NA 0.6 designs with varying sizes of metalens: 6.75 μm, 12.5 μm (reference from Figure 8), 25 μm, and 50 μm, as shown in Figures 10A–10D. Figure 10E shows focusing efficiencies between 70% and 80%, with smaller metalenses achieving greater efficiencies. The design-time analysis in Figure 10F reveals linear scaling that depends solely on the simulation size. This demonstrates that our proposed method maintains computational efficiency even with increased design variables, with simulation size being the primary factor affecting design time. Additionally, the average efficiency analysis shows an inverse relationship with metalens size, consistent with findings from previous studies.^38^^,^^83^^,^^84^

Our proposed method can also be extended to fabrication-compatible structures by modifying the design variables. For example, grating-based configurations can be implemented by averaging the gradient of design variables along the vertical direction to enforce structural uniformity and enhance lithographic feasibility.^38^^,^^85^

### Cross-validation of in-house FDTD code

**Figure 11:**
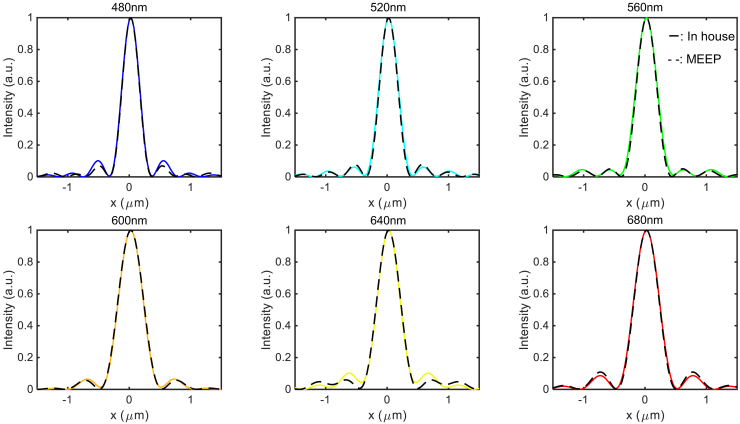
Comparison of focal plane intensity distributions obtained from our in-house FDTD (solid lines) and MEEP (dashed lines) at representative wavelengths ranging from 480 nm to 680 nm The design corresponds to a metalens with NA = 0.6.

Before drawing final conclusions, we verified the accuracy of our in-house FDTD code by cross-validating it against the open-source electromagnetic solver based on FDTD, MIT electromagnetic equation propagation (MEEP). As a benchmark, we selected a metalens design with a numerical aperture (NA) of 0.6. In Figure 11, the focal field profiles and focusing efficiencies obtained from both our in-house code and MEEP were in close agreement across the entire visible spectrum, confirming the reliability and consistency of our simulation results. This validation step reinforces the robustness of our methodology and ensures that the reported performance metrics are accurate and reproducible across independent simulation platforms.

## Discussion and conclusion

This study presents a dynamic pulse-modulated time-domain adjoint optimization technique that addresses the limitations of both frequency-domain and conventional time-domain adjoint methods in achromatic metalens design. By dynamically adjusting incident pulses during the optimization process, the proposed method tackles critical challenges, including inefficiencies caused by frequency sampling in frequency-domain adjoint methods and non-uniform spectral performance in conventional time-domain adjoint methods. This proposed approach ensures consistent simulation times regardless of the frequency count, delivering significant enhancements in both average efficiency and spectral uniformity across the band of interest.

Using the proposed methodology, we successfully designed achromatic metalenses with varying NAs and lens sizes, demonstrating high efficiency and near–uniform spectral responses with reduced the design time compared to frequency-domain adjoint method. Our comprehensive investigation revealed exceptional performance metrics: for NA 0.99, absolute focusing efficiency improved by 20%–30% compared with frequency-domain designs and improved by 10%–15% compared with conventional time-domain designs with a near-uniform response. Average efficiency increased from 27% to 45%. For lower-NA designs, we achieved FWHM values and field distributions approaching the diffraction limit. Scalability analysis highlighted linear scaling of simulation time with design size, while maintaining focusing efficiencies of 70%–80%.

Our dynamic pulse-modulated time-domain adjoint method demonstrated a robust optimization framework for achromatic metalens designs. Unlike frequency-domain adjoint approaches, which require extensive simulations for each sampled frequency, our method significantly reduces simulation time while achieving superior optical performance. Under the given design objective function and simulation dimensions, the proposed method shows simulation times of 4.98 s per iteration compared to 61.8 s for frequency domain methods—demonstrating its computational efficiency. Furthermore, the proposed method achieves near-diffraction-limited focusing with uniform spectral response in NA 0.6 and NA 0.4, overcoming the trade-offs inherent in conventional approaches. These advances make our methodology a powerful design tool in nanophotonics, enabling high-performance, broadband optical devices.

### Limitations of the study

This study, based on time-domain adjoint optimization, has inherent computational challenges. First, the method requires storing both the forward and adjoint electric fields at every time step for the entire design domain. This increases memory demands over the FDTD time stepping, unlike the frequency-based adjoint optimization method. Second, our time-domain adjoint method cannot exploit near-to-far field transformations. Therefore, full-wave simulations for space, including the focal plane, are required. This limits the scalability of our method in some of the applications.

Future work may explore memory-efficient gradient computation techniques or integrate hybrid methods to reduce computational overhead while preserving the benefits of time-domain broadband optimization.

## Resource availability

### Lead contact

Requests for further information and resources should be directed to and will be fulfilled by the lead contact, Kyung-Young Jung (kyjung3@hanyang.ac.kr).

### Materials availability

This study did not use or generate any physical materials.

### Data and code availability

• The datasets generated and/or analyzed during this study are available from the lead contact upon reasonable request.
• The codes used in this study are available from the lead contact upon reasonable request.
• Any additional information required to reanalyze the data reported in this paper is available from the lead contact upon request.

## Acknowledgments

This work was supported by the 10.13039/501100003725National Research Foundation of Korea (NRF) grant funded by the Korean government (MSIT) (RS-2024-00409492, RS-2024-00338048) and also supported by Culture, Sports and Tourism R&D Program through the Korea Creative Content Agency grant funded by the 10.13039/501100003561Ministry of Culture, Sports and Tourism in 2024(RS-2024-00332210) and under the artificial intelligence semiconductor support program to nurture the best talents ((IITP-(2025)-RS-2023-00253914) grant funded by the Korea government. The authors thank all members of the lab for their support.

## Author contributions

Conceptualization, M.P., H.C., and K.-Y.J.; methodology, M.P., H.C., and K.-Y.J.; investigation, M.P.; writing – original draft, M.P.; writing-review and editing, H.C. and K.-Y.J.; funding acquisition, H.C. and K.-Y.J.; supervision, K.-Y.J. and H.C.

## Declaration of interests

The authors declare no competing interests.

## STAR★Methods

### Key resources table

**Table:** 

REAGENT or RESOURCE	SOURCE	IDENTIFIER
**Software and algorithms**		

MATLAB	MathWorks Co., LTD	https://www.mathworks.com/products/matlab.html
Visual Studio 2022	Visual Studio 2022	https://visualstudio.microsoft.com/

### Method details

#### FDTD solver implementation

We implemented a custom finite-difference time-domain (FDTD) solver in C++ to simulate electromagnetic wave propagation. Maxwell’s curl equations were discretized on a uniform Yee grid with a spatial resolution of Δx=Δy=Δz=25nm. The temporal resolution Δt was determined by the Courant–Friedrichs–Lewy (CFL) stability criterion, using a Courant number of S=cΔt/Δx=0.99. To prevent non-physical reflections at the domain boundaries, perfectly matched layers (PMLs) with a thickness of 10 grid cells were applied on all sides.

#### Design region and parameterization

The metasurface design region was defined over a grid of $N_{x}\times N_{y}$ cells, corresponding to the metalens aperture (e.g., 500 × 500 cells for a 12.5 μ m diameter lens). The relative permittivity at each cell, $\varepsilon_{r}\left(r\right)$, was parameterized by a continuous design variable ρ(r)∈[0,1] and interpolated as

$$\varepsilon_{r}\left(\rho \right)=\varepsilon_{\text{air}}+\left(\varepsilon_{{\text{TiO}}_{2}}-\varepsilon_{\text{air}}\right)\;\rho \left(r\right)\text{,}$$

where $\varepsilon_{{\text{TiO}}_{2}}=6$ and $\varepsilon_{\text{air}}=1$.

#### Forward simulation

A normally incident plane wave was introduced using the total-field/scattered-field (TF/SF) method. The incident field was a broadband pulse defined as

$$s\left(t\right)=\text{sinc}\left[2\pi f_{\text{BW}}\left(t-t_{0}\right)\right]\cos\left(2\pi f_{0}t\right)\text{,}$$

windowed by a Hamming envelope of 100 fs duration, covering a spectral range from 470 nm to 700 nm. The electromagnetic fields were recorded both at the focal plane and across the design region to enable subsequent gradient computation.

#### Adjoint simulation and gradient evaluation

At each optimization step, the objective function

$$F=\frac{1}{2}\int_{0}^{T_{f}}{\left|E_{z}\left(t,r_{\text{focus}}\right)\right|}^{2}\;dt$$

was computed as the time-integrated field intensity at the focal point $r_{\text{focus}}$. The adjoint source was constructed by time-reversing the recorded $E_{z}$ field at the focal point, and a second FDTD simulation was performed to propagate this source backward through the design domain. Gradients with respect to the design variable ρ(r) were obtained via the correlation of forward and adjoint fields:

$$\frac{\partial F}{\partial \rho \left(r\right)}=-\left(\varepsilon_{{\text{TiO}}_{2}}-\varepsilon_{\text{air}}\right)\;\varepsilon_{0}\int_{0}^{T_{f}}\frac{\partial E_{z}\left(t,r\right)}{\partial t}\;E_{\text{adj},z}\left(t,r\right)\;dt\text{.}$$

#### Optimization algorithm

We employed gradient descent to optimize the design variables. To promote binary patterns, we introduced a penalization term:

$$R\left(\rho \right)=\beta \sum_{r}\rho \left(r\right)\left[1-\rho \left(r\right)\right]\text{,}$$

where the regularization weight *β* was gradually increased over iterations. The optimization proceeded for 300 steps or until convergence of the figure of merit.

#### Broadband and spectral modulation strategy

The initial optimization phase used the full-bandwidth sinc pulse to ensure broadband response. After 50 iterations, spectral regions with poor focusing performance were identified by analyzing the instantaneous focal efficiency spectrum. In subsequent iterations, narrowband Gaussian-modulated pulses, centered at the underperforming frequencies, were alternated to enhance spectral uniformity:

$$s_{\text{Gauss}}\left(t\right)=\exp\;\left[-\frac{{\left(t-t_{0}\right)}^{2}}{2\sigma^{2}}\right]\cos\left(2\pi f_{\min}t\right)\text{.}$$

#### Post-processing and performance metrics

All post-processing was performed in MATLAB R2022a. Key performance metrics included the focusing efficiency, defined as the energy within the first intensity minimum normalized by the total incident energy. Additional metrics such as the full-width at half-maximum (FWHM), focal length, and spectral uniformity were extracted directly from the simulated field distributions.

### Quantification and statistical analysis

No statistical analyses were performed; all results derive from deterministic electromagnetic simulations.
